# Intrahepatic bile duct exploration lithotomy is a useful adjunctive hepatectomy method for bilateral primary hepatolithiasis: an eight-year experience at a single centre

**DOI:** 10.1186/s12893-019-0480-1

**Published:** 2019-02-04

**Authors:** En-liang Li, Rong-fa Yuan, Wen-jun Liao, Qian Feng, Jun Lei, Xiang-bao Yin, Lin-quan Wu, Jiang-hua Shao

**Affiliations:** 1 Department of Hepatobiliary and Pancreatic surgery, the Second Affiliated Hospital of Nanchang University, Nanchang, China; 2 Zhejiang University school of Medicine, HangZhou, China

**Keywords:** Intrahepatic bile duct, Hepatectomy, Bilateral hepatolithiasis, Treatment

## Abstract

**Background:**

To evaluate the perioperative and long-term results of intrahepatic bile duct exploration lithotomy (IHBDIL) combined with hepatectomy for patients with complicated bilateral primary hepatolithiasis.

**Methods:**

A study was conducted involving 56 patients with complicated bilateral primary hepatolithiasis who underwent IHBDIL combined with hepatectomy at our hospital from January 2006 to December 2014. The perioperative and long-term outcomes that were retrospectively analysed included the stone clearance rate, operative morbidity and mortality, and stone recurrence rate. Patients with a preoperative diagnosis of cholangiocarcinoma were excluded.

**Results:**

In all 56 patients, hepatic duct stones were located in the bilateral IHBD. The surgical method was IHBDIL combined with hepatectomy. Postoperative complications occurred in 15 patients (26.8%), 14 patients responded to conservative management, and there was 1 case of postoperative mortality because of hepatic failure. The overall initial success rate of stone clearance was 85.7%, and the final clearance rate was 92.9% following postoperative choledochoscopic lithotripsy. The stone recurrence rate was 13.5%, and the occurrence of postoperative cholangitis was 10.9% during the follow-up period.

**Conclusion:**

IHBDIL combined with hepatectomy is a safe, effective, and promising treatment for patients with complicated bilateral primary hepatolithiasis. The perioperative and long-term outcomes are satisfactory for complicated bilateral primary hepatolithiasis.

**Electronic supplementary material:**

The online version of this article (10.1186/s12893-019-0480-1) contains supplementary material, which is available to authorized users.

## Background

Primary hepatolithiasis is defined as the presence of stones in the intrahepatic bile ducts (IHBDs); this condition is endemic in the Asia-Pacific region and is becoming increasingly common in Western populations [1]. Complicated hepatolithiasis could be extended to include complications of intrahepatic strictures and bilateral stones [2]. Complicated hepatolithiasis can easily lead to biliary obstruction, recurrent biliary tract infection, biliary liver abscessation, biliary cirrhosis and cholangiocarcinoma. The current treatment methods mainly include hepatectomy combined with common bile duct (CBD) exploration [3].

However, because patients with complicated bilateral hepatolithiasis have perihepatic extensive adhesions from previous operations, recurrent biliary tract infections, liver abscesses and other complications, hepatectomy can become particularly difficult, and there is a higher residual stone rate. In addition, complicated bilateral hepatolithiasis can affect multiple hepatic segments and lead to hepatic parenchymal atrophy. Complete clearance of this type of IHBD stone requires resection of multiple liver segments; however, patients with complicated bilateral hepatolithiasis often cannot tolerate resection of multiple liver segments, which could easily lead to postoperative hepatic failure. Therefore, the treatment of complicated bilateral hepatolithiasis remains a challenge.

Our previous study found that IHBD exploration lithotomy for complex IHBD stones achieved satisfactory results [4]. To this end, this study used IHBDIL combined with partial hepatectomy for the treatment of complicated bilateral hepatolithiasis to further assess the safety and efficacy of this treatment method.

## Methods

### Patients

Our department treated 4586 patients with hepatolithiasis from January 2006 to December 2014. The study included 56 patients with stones mainly located in the bilateral IHBD; these patients underwent IHBDIL combined with hepatectomy. The inclusion criteria for this study were as follows: (1) the stones were located in multiple segments of the liver; (2) the patients could not tolerate multiple segmental resection; (3) the distal bile duct where the stones were located was dilated, and the proximal bile duct did not have severe stricture; and (4) patients had Child-Pugh class A or B disease. The exclusion criteria for this study were as follows: (1) the stones could be removed by routine hepatectomy and CBD exploration; (2) the patients were highly suspected of having cholangiocarcinoma; (3) the patients could not tolerate surgery due to poor general conditions; or (4) the patients refused surgery. Informed consent for the surgical procedures was obtained from each patient. This study was approved by the Ethics Committee of the Second Affiliated Hospital of Nanchang University.

### Preoperative evaluation

All patients were evaluated with liver function testing, preoperative ultrasound (US), contrast-enhanced computed tomography (CT), magnetic resonance imaging (MRI) or magnetic resonance cholangiopancreatography (MRCP). These examinations provided information regarding the locations of the stones and characterized the patient’s biliary system anatomy and lesion pathology. In addition, percutaneous transhepatic cholangiography (PTC) or endoscopic retrograde cholangiopancreatography (ERCP) was performed selectively for patients with IHBD dilatation; these procedures are aimed at delineating the site of the bile duct stricture and improving liver function through bile drainage. In all cases of complicated bilateral hepatolithiasis, a volumetric CT scan was performed, and an indocyanine green 15-min retention rate (ICG-R15) was measured to estimate the volume of the remnant liver and to determine the type of surgery.

### Surgical procedure

Surgery was performed through a right subcostal incision with midline upward extension to the xiphoid process. We routinely perform cholecystectomy and free livers, and the use of US and choledochoscopy during surgery also helped us to further understand the positions and sizes of the stones and the conditions of the bile duct (strictured or dilatated).

**Fig. 1:**
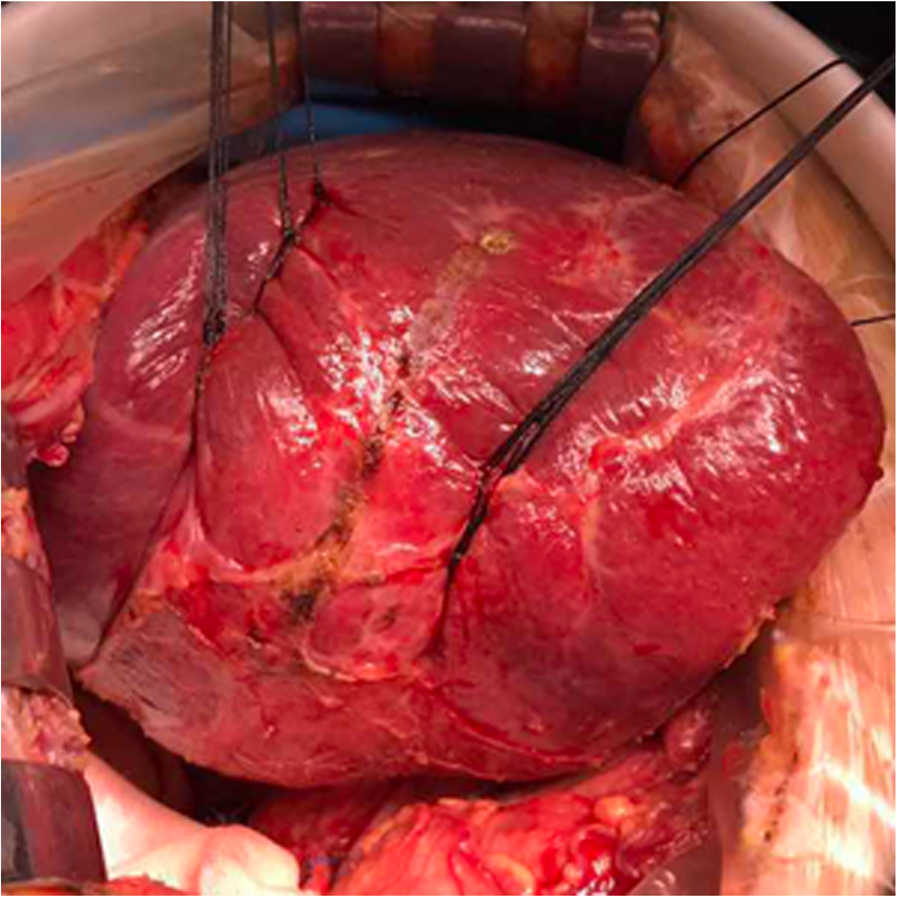
Taking the diseased bile duct as the centre, several sutures were sewn on both sides to provide as intraoperative traction

**Fig. 2:**
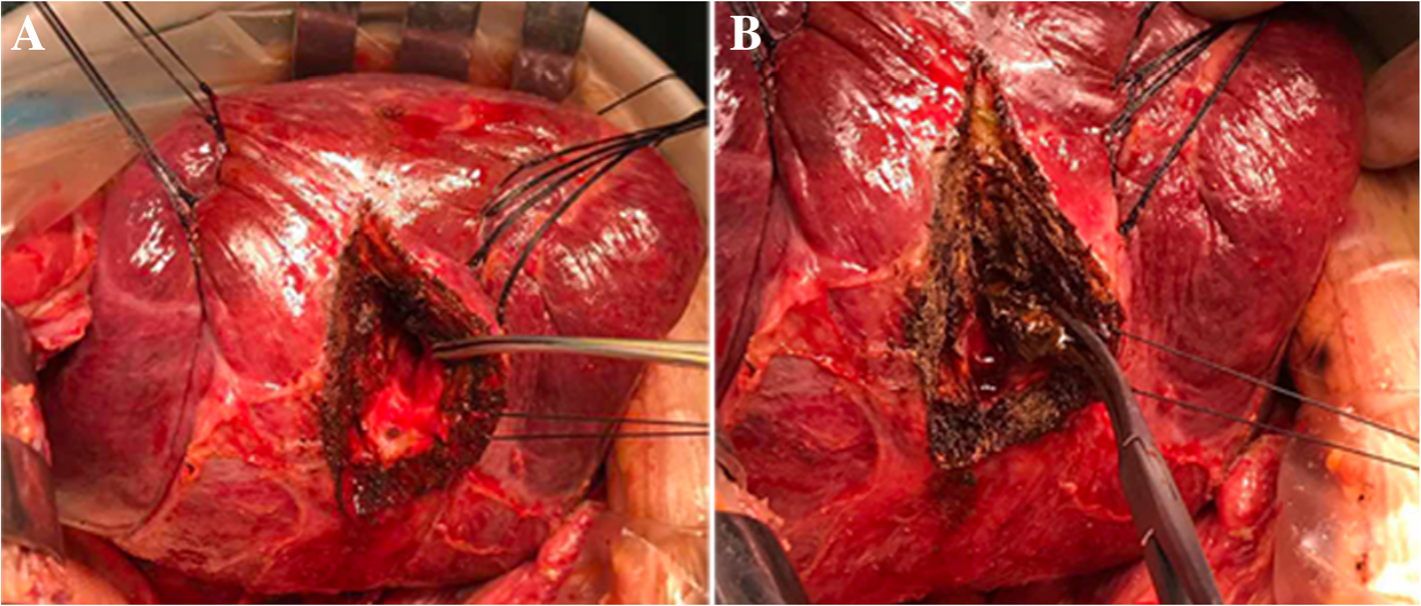
**a** An incision was made on the surface of the liver parenchyma where the calculus was located to expose and incise the dilated intrahepatic bile duct approximately 1.0 cm. **b** The stones were completely removed from the diseased region to the hepatic portal bile duct and distal IHBD and its branch

**Fig. 3:**
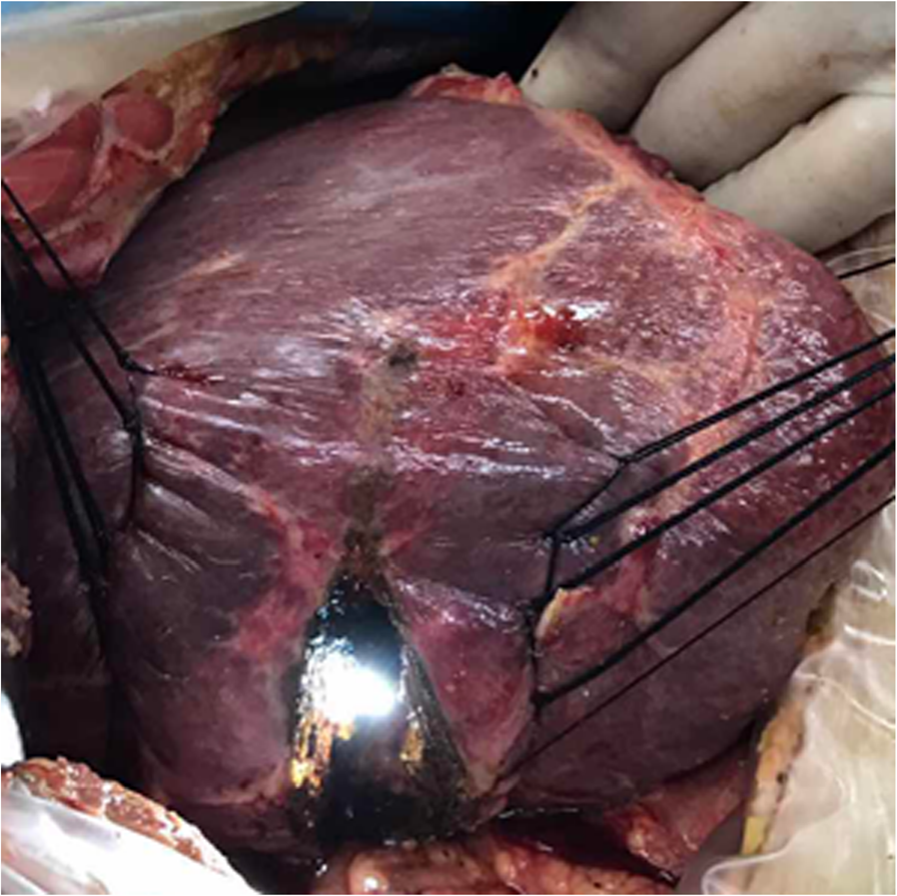
Choledochoscopy was performed again to confirm no obstruction between the diseased IHBD and the hepatic hilar bile duct or CBD

**Fig. 4:**
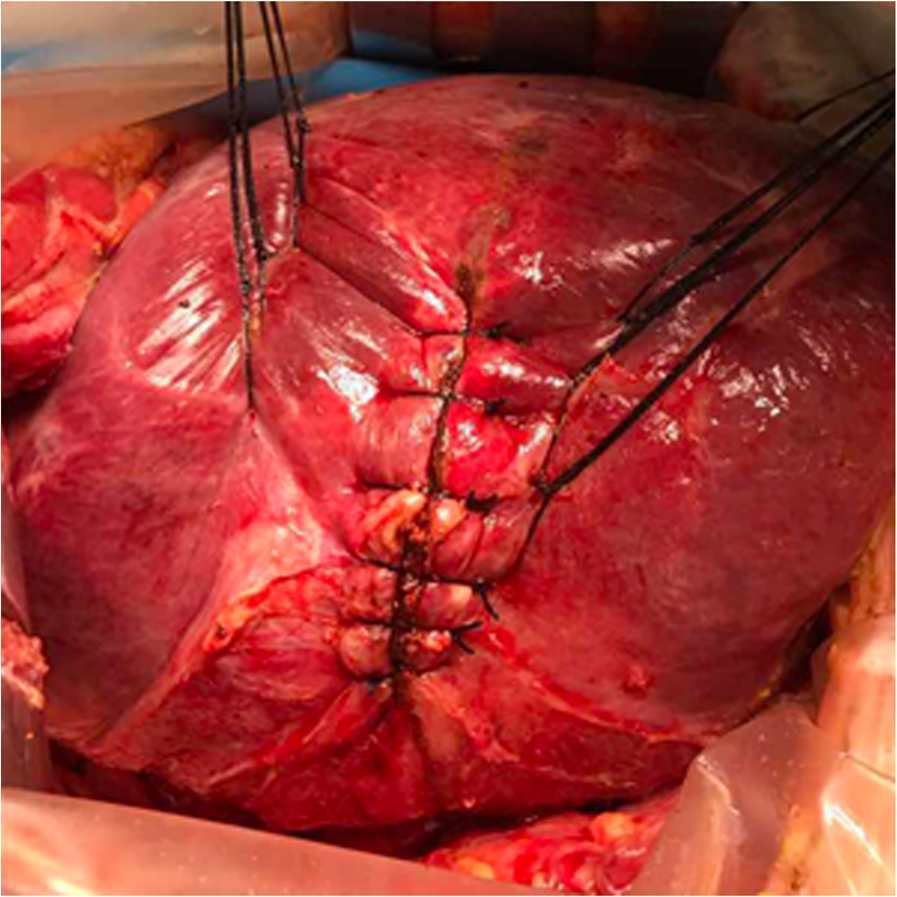
The hepatic bile duct and surface of the liver parenchyma were sutured

Hepatic segments with severe stricture of the IHBD or parenchymal atrophic change should be resected. Hepatectomy was performed using a clamp crushing method. We applied the Pringle manoeuvre to occlude the blood inflow to the liver if necessary. CBD exploration was performed to remove stones in the extrahepatic bile duct. Furthermore, we could continue to remove stones in the IHBD via the CBD with choledochoscopy and a stone basket. When the stones could not be removed due to a location in the peripheral IHBD or when the stones were arranged in a beaded manner and incarcerated in the IHBD, we had to use IHBDIL with no or mild stricture of the IHBD and without parenchymal atrophic change or liver fibrosis. During IHBDIL, we could touch the liver parenchyma with our fingers to roughly explore the location of calculi in the hepatic segments. In addition, we located the positions of the stones in the diseased bile duct with intraoperative US. First, taking the diseased bile duct at the centre, we sewed traction sutures on two sides of the diseased bile duct on the surface of the liver parenchyma (Fig. 1). These intraoperative traction sutures were helpful in exposing the IHBD and reducing bleeding. Through real-time intraoperative US monitoring, we could avoid damaging the major intrahepatic blood vessels. Second, to expose stones in the IHBD, we incised in an anterograde incision on the surface of the liver parenchyma. We opened the dilated IHBD approximately 1.0 cm and removed the stones completely from the diseased IHBD to the hepatic portal bile duct with forceps, Fogarty catheters, or saline flushing (Fig. 2). Third, choledochoscopy was performed to confirm that there were no obstructions or residual stones between the diseased IHBD or its branches and the hepatic hilar bile duct or CBD (Fig. 3) and to assess the initial stone clearance rate. Finally, we used 5–0 Prolene non-absorbable suture to continuously close the IHBD. We then employed silk thread to close the surface of the liver parenchyma discontinuously (Fig. 4) and removed the traction sutures. We placed a “T” tube for external drainage after choledocholithotomy. The detailed surgical procedure is shown in Additional file 1: Table S1.

### Follow-up

The follow-up period lasted until December 2015, and the postoperative follow-up began 1 month after surgery. All patients were followed for routine liver function tests and recurrence of the stones by US or CT monitoring every 3 months. MRCP or ERCP was performed whenever the patients presented with symptoms suggestive of cholangitis. In this study, one patient died of hepatic failure due to sudden severe hepatitis and secondary biliary cirrhosis one week after surgery. At the end of the study, 55 of 56 patients completed a median follow-up period of 52 months (12–72 months). The perioperative outcomes included the stone clearance rate and operative morbidity and mortality, and the long-term outcomes included the stone recurrence rate and recurrence of attacks of acute cholangitis.

### Statistical analysis

Data were analysed retrospectively. The perioperative outcomes included the stone clearance rate, operative morbidity, and mortality. The long-term outcomes included the stone recurrence rate and acute cholangitis. Continuous data are expressed as the mean ± standard deviation (SD).

## Results

### Patient characteristics

There were 21 men and 35 women with an average age of 53.6 years (range, 34 to 78 years). The main symptoms of patients with hepatolithiasis were abdominal pain, fever, and jaundice. Twenty-three (41.1%) patients had previously undergone one or more biliary operations. Twenty-four (42.9%) patients also had extrahepatic stones. Nine (16.1%) patients also had liver cysts, and 13 (23.2%) had biliary cirrhosis. Thirteen (23.2%) patients had a history of chronic hepatitis B virus (HBV), and 22 (39.3%) had chronic diseases, such as hypertension (6/56), coronary heart disease (7/56), or diabetes (9/56). The locations of the stones and operative procedures are summarized in Table 1.

**Table 1:** Distribution of the Stones and Operative procedures

Stone Location	No. Patients(*n* = 56)	Site of Biliary Stricture
S_2_, S_3_, S_8_	8 (14.3%)	Left lateral sectional duct
S_2_, S_4_, S_8_	7 (12.5%)	Left hepatic duct
S_2_, S_4_, S_6_ + CBD	7(12.5%)	Left lateral sectional duct,VI sectional duct
S_3_, S_4_, S_7_	6 (10.3%)	Left hepatic duct
S_3_, S_5_, S_8_	7 (12.5%)	Left lateral sectional duct,Vsectional duct
S_3_, S_4_, S_8_ + CBD	8 (14.3%)	Left hepatic duct
S_2_, S_3_, S_7_, S_8_ + CBD	4 (7.1%)	Left lateral sectional duct
S_2_, S_4_, S_6_, S_8_	4 (7.1%)	Left lateral sectional duct,VI sectional duct
S_2_, S_4_, S_5_, S_8_ + CBD	5 (8.9%)	Left lateral sectional duct,Vsectional duct

### Perioperative outcome

The intraoperative and postoperative results are listed in Table 2. The mean operative time was 252.3 ± 8.7 min. The mean intraoperative blood loss was 586.4 ± 85.3 mL. The mean duration of the postoperative hospital stay was 13.7 ± 5.4 days. Postoperative complications were observed in 15 patients (26.8%). The most common complication was bile leakage, followed by wound infection. According to the modified Clavien classification [5], there were 8 cases of grade I complications, 3 cases of grade II complications, 3 cases of grade IIIa complications, and 1 case of a grade V complication. All complications except the grade V complication improved by the time of discharge. Patients’ liver function parameters gradually returned to normal serum concentrations during hospitalization (Additional file 2: Table S2). However, 2 patients with grade IIIa bile leakage had two times the normal level of alanine aminotransferase (ALT) discharge, without fever or jaundice. These two patients were treated with hepatoprotective drugs after discharge, and their ALT levels returned to normal after one month of follow-up. There was 1 death due to hepatic failure during the postoperative period. One patient with HBV infection experienced sudden severe hepatitis and secondary biliary cirrhosis and underwent hepatectomy (left lateral lobectomy, S_5_) plus IHBDIL (S_4_, S_8_) and choledocholithotomy. This patient experienced bile leakage and sustained a high fever and progressive liver function decline postoperatively. This patient died on the seventh postoperative day due to multiorgan failure caused by hepatic failure. Seven patients had different degrees of liver atrophy at the site of the IHBDIL liver segments, but their liver function was normal.

**Table 2:** Perioperative Outcome

Variable	
Duration of operation (min) ± SD	252.3 ± 68.7
Intraoperative blood loss (ml) ± SD	586.4 ± 85.3
Intraoperative transfusion, n (%)	21 (37.5%)
Postoperative hospital stay (day) ± SD	13.7 ± 5.4
Postoperative complications, n (%)	15(26.8%)
Bleeding	0
Pleural effusion	3(5.4%)
wound infection	2(3.6%)
Intraabdominal fluid collection	3(5.4%)
Intraabdominal hematoma	1(1.8%)
Biliary leakage	5(8.9%)
Hepatic failure	1(1.8%)
Clavien-Dindo classification of surgical complications, *n* (%)	
Grade I	8(14.3%)
Pleural effusion	3(5.4%)
Wound infection	2(3.6%)
Intraabdominal fluid collection	3(5.4%)
Grade II	3(5.4%)
Biliary leakage	3(5.4%)
Grade IIIa	3(5.4%)
Biliary leakage	2(3.6%)
Intraabdominal hematoma	1(1.8%)
Grade IV	0
Grade V	
Death due to Hepatic failure and sepsis	1(2.9%)

### Outcome of stone clearance

At the end of this study, the initial success rate of stone clearance was 85.7% (48 patients), and the final clearance rate was 92.9% (52 patients) following postoperative choledochoscopic lithotripsy. Fifty-five patients completed the follow-up, including 52 whose stones were completely removed at the initial surgery and three patients with residual stones. With a median follow-up of 52 months, recurrent stones developed in 7 (13.5%) of 52 patients who had no residual stones. The rate of acute cholangitis was 10.9% (6/55). Stone recurrence and residual stones were the major causes of postoperative acute cholangitis. The details of these patients are shown in Table 3.

**Table 3:** Outcome of Stone Clearance

Variable	No. of patients
Initial clearance rate ^a^	48/56 (85.7%)
Final clearance rate ^b^ after postoperative ERCP, EST	52/56 (92.9%)
Recurrent stone	7/52 (13.5%)
Recurrence attack of acute cholangitis	6 /55(10.9%)

^a^Initial clearance was defined as clearance of stones immediately postoperatively.atus

^b^Final clearance was defined as clearance of stones at discharge

*ERCP* endoscopic retrograde cholangiopancreatography, *EST* endoscopic sphincterotomy

## Discussion

Hepatolithiasis is defined as the presence of gallstones in the bile ducts proximal to the confluence of the right and left hepatic ducts, irrespective of the co-existence of gallstones in the common bile duct and/or gallbladder [1]. The main aims of definitive surgery for primary hepatolithiasis are to relieve abdominal pain, eliminate future attacks and prevent disease progression. These goals are achieved by conducting scientific treatment using a multidisciplinary approach [6]. Nonoperative treatments are a feasible option and are mainly suitable for patients with mild and less extensive forms of hepatolithiasis or for some elderly patients who are unable to tolerate surgery [7].

Hepatectomy remains the main treatment option for hepatolithiasis. Hepatic resection for hepatolithiasis has generally been considered for patients with unilateral disease because this method can be used to completely remove the stones, remove the lesion, relieve the obstruction, smooth the drainage, and reduce the recurrence of stones [8, 9]. However, this procedure remains difficult when patients present with bilateral hepatolithiasis [10]. A study from Japan reported performing unilateral hepatectomy in 7 patients with bilateral hepatolithiasis, and 5 of these patients experienced residual stones postoperatively [11]. In addition, there are very limited data addressing the management of bilateral hepatolithiasis [12]. One study found that extrahepatic bile duct exploration was associated with a significantly higher overall complication rate, longer hospital stay, higher rate of residual stones and stone recurrence than unilateral hepatectomy combined with bile duct exploration to treat bilateral hepatolithiasis [13]. Some authors believe that bilateral hepatectomy for bilateral hepatolithiasis is feasible; however, with the prerequisite of sufficient remnant liver, the surgical requirements are very stringent [14]. In addition, in patients with complicated bilateral hepatolithiasis, atrophy and/or anatomical changes are present in the hepatic parenchyma, forming an atrophy–hypertrophy complex and causing a posterior medial rotation and translocation of the vena cava inferior to the first hepatic portal and hepatic segment/interlobular fissure [15]. Therefore, even though patients may have enough remnant liver function, bilateral hepatectomy increases intraoperative bleeding and the risks of surgery for complicated bilateral hepatolithiasis [16].

Our technique of IHBDIL was based on the experiences of choledochotomy and CBD exploration for treating CBD stones and adaptions using intraoperative choledochoscopy for treating IHBD stones. IHBDIL with intraoperative US guidance can be used to accurately incise the expansion of the IHBD and, when used in combination with choledochoscopy, improves the rate of stone clearance. To the best of our knowledge, our series is the only series to evaluate the efficacy of IHBDIL combined with hepatectomy for bilateral primary hepatolithiasis. The majority of our patients underwent multiple surgeries, had bilateral liver segment stones, and had poor liver function; thus, their liver function depended on hypertrophy of the liver segment. In this situation, if we had used bilateral hepatectomy to address complicated hepatolithiasis, the remnant liver would have struggled to maintain liver function and thus increase the risk of surgery. In addition, we confirmed that IHBD exploration lithotomy involving cutting open the liver surface to treat complex hepatolithiasis is safe and feasible. This study adopted IHBDIL combined with hepatectomy as a surgical method and obtained satisfactory results. First, the stone clearance rate after surgery was 85.7%, and the final clearance rate was 92.9% following postoperative choledochoscopic lithotripsy. The stone recurrence rate was 13.5%, and the occurrence of postoperative cholangitis was 10.9% after a median follow-up period of 52 months. Our results were similar to those reported in most studies after hepatic resection or bilateral resection [12, 14, 17]. These findings indicated that the long-term outcomes of IHBDIL combined with hepatectomy for complicated hepatolithiasis were satisfactory. Second, the most common postoperative complication was bile leakage (8.9%), followed by intra-abdominal fluid collection and pleural effusion (5.4%) and wound infection (2.9%). Compared with the results published in previous literature, our current data showed similar outcomes for postoperative complications [2, 14, 16, 18–20]. Therefore, IHBDIL could be a good and safe assisted hepatectomy option as a minimally invasive treatment for bilateral hepatolithiasis.

Although our study showed that IHBDIL combined with hepatectomy achieved satisfactory clinical results, we should recognize that this surgical approach has some limitations, as do other types of surgery. The effectiveness and thoroughness of hepatectomy must be fully appreciated to treat hepatolithiasis. However, in the following cases, IHBDIL could be considered to assist hepatectomy: (1) Stones are mainly located in the bilateral liver or multiple liver segments, or patients with serious liver function damage and secondary biliary cirrhosis cannot tolerate multiple hepatic resection. (2) The stone is located in the peripheral IHBD or in a beaded arrangement incarcerated in the IHBD, or CBD exploration and cholangioscopy cannot remove distal stones of the IHBD. However, IHBDIL also has the following contraindications: (1) preoperative and intraoperative confirmation of severe IHBD stricture, and (2) liver parenchymal lesions with atrophy and fibrosis. Furthermore, in this study, 6 of 15 patients with postoperative complications underwent IHBDIL in two IHBDs, 1 patient died, 2 patients developed grade IIIa bile leakage, 1 patient had an intra-abdominal haematoma, 1 patient had an intra-abdominal fluid collection, and 1 patient developed a pleural effusion. The incidence of postoperative complications was as high as 40% (6/15). Therefore, to reduce serious postoperative complications, we recommend cautious use of IHBDIL in two or more IHBDs.

## Conclusions

Our study demonstrated that IHBDIL combined with hepatectomy is a safe and effective procedure for selected patients with complicated bilateral hepatolithiasis. However, this study involved a relatively small number of patients and was performed at a single medical centre. We recommend that future studies with an increased number sample size be performed to provide evidence-based support for our current observations.

## Additional files

Additional file 1: **Table S1.** Operative Procedures. (DOCX 14 kb)

Additional file 2: **Table S2.** Postoperative liver function. (DOC 14 kb)

## Abbreviations

CBD: Common bile duct
IHBD: Intrahepatic bile duct
IHBDIL: Intrahepatic bile duct exploration lithotomy
