# Supplementary material for: Intrahepatic bile duct exploration lithotomy is a useful adjunctive hepatectomy method for bilateral primary hepatolithiasis: an eight-year experience at a single centre
Source: BMC Surg. 2019 Feb 4;19:16. doi: 10.1186/s12893-019-0480-1 (PMC6360740; doi:10.1186/s12893-019-0480-1)
Supplement: Supplementary file 1 — Table S1. Operative Procedures. (DOCX 14 kb) [file 12893_2019_480_MOESM1_ESM.docx]

| Additional file 1: **Table S1**. Operative procedures | |
| --- | --- |
| Operative procedures | No. Patients(n=56) |
| Hepatectomy(Left lateral lobectomy)+ IHDIL (S_8_) | 8 (14.3%) |
| Hepatectomy(Left hepatectomy)+ IHDIL (S_8_) | 7 (12.5%) |
| Hepatectomy(Left lateral lobectomy,S_6_)+IHDIL(S_4_)  +Choledocholithotomy | 7(12.5%) |
| Hepatectomy(Left hepatectomy)+ IHDIL (S_7_) | 6 (10.3%) |
| Hepatectomy(Left lateral lobectomy, S_5_)+ IHDIL (S_8_) | 7 (12.5%) |
| Hepatectomy(Left hepatectomy)+ IHDIL (S_8_) + Choledocholithotomy | 8 (14.3%) |
| Hepatectomy(Left lateral lobectomy)+IHDIL(S_7,_S_8_)  + Choledocholithotomy | 4 (7.1%) |
| Hepatectomy(Left lateral hepatectomy, S_6_)+ IHDIL (S_4,_S_8_) | 4 (7.1%) |
| Hepatectomy(Left lateral lobectomy,S_5_)+IHDIL(S_4,_S_8_)  + Choledocholithotomy | 5 (8.9%) |
