# Supplementary material for: Intrahepatic bile duct exploration lithotomy is a useful adjunctive hepatectomy method for bilateral primary hepatolithiasis: an eight-year experience at a single centre
Source: BMC Surg. 2019 Feb 4;19:16. doi: 10.1186/s12893-019-0480-1 (PMC6360740; doi:10.1186/s12893-019-0480-1)
Supplement: Supplementary file 2 — Table S2. Postoperative liver function. (DOC 14 kb) [file 12893_2019_480_MOESM2_ESM.doc]

| Additional file 2: Table S2. Postoperative liver function | | | |
| --- | --- | --- | --- |
| Characteristic | First day | Third day | Sixth day |
| ALB(g/L) | 31.35±5.21 | 33.27±3.42 | 38.36±2.86 |
| ALT(U/L) | 87.38±26.64 | 63.26±18.76 | 36.18±5.78 |
| TBIL(μmol/L) | 96.75±36.85 | 82.56±28.47 | 40.21±8.63 |

ALB, albumin; ALT, alanine aminotransferase; DBIL, direct bilirubin
